# Supplementary material for: Essential Functions of Calmodulin and Identification of Its Proximal Interacting Proteins in Tachyzoite-Stage Toxoplasma gondii via BioID Technology
Source: Microbiol Spectr. 2022 Oct 10;10(5):e01363-22. doi: 10.1128/spectrum.01363-22 (PMC9602672; doi:10.1128/spectrum.01363-22)

Fig S1. Failure in CaM direct knockout and CaM knockdown using tetracycline-repressive sag1 promoter. (A) The construction pattern of CaM directly knockout system through CRISPR-Cas9 technology in RH△*hxgprt* parent strain. Diagnostic PCRs on the pool were selected with pyrimethamine. (B) Plaque assay of iCaM without ty tag pretreated with or without ATc for ten days. (C) Unsuccessful construction of CaM knockdown system using tetracycline-repressive sag1 promoter. Two clones obtained here lost sag1 promoter and ty tag. (D) Multiple alignments of part DNA sequences from two clones without ty tag. The multiple alignment program for nucleotide sequences was MAFF version 7 with the G-INS-1 strategy and the unaligned-level 0.8.

Fig S2. Plaque of iCaM pretreated with or without ATc.

Fig S3. Daughter tachyzoite division orientation in parasites upon CaM depletion. (A) IFA of parasites stained with rabbit anti-IMC1 to show the daughter tachyzoite. Scale bar 5 μm. (B) Division orientations of daughter tachyzoite in PV. Means ± SD of three independent experiments. **** $p<0.0001$, two-way ANOVA with Tukey's multiple comparison test.

Fig S4. Amylopectin accumulation in parasites upon CaM depletion. The parasites of iCaM and TATI were grown on coverslips for 48 h in the presence or absence of ATc and then fixed with PFA for PAS staining.

Fig S5. Interaction between CaM and MyoF revealed by CaM-BioID and Co-IP. (A) The unique peptide counts of Myosin F and Myosin J were identified in CaM-BioID data. (B) Co-IP between CaM and MyoF in *T. gondii* tachyzoite. (C) Co-IP between CaM and MyoJ in *T. gondii* tachyzoite.

Fig S1

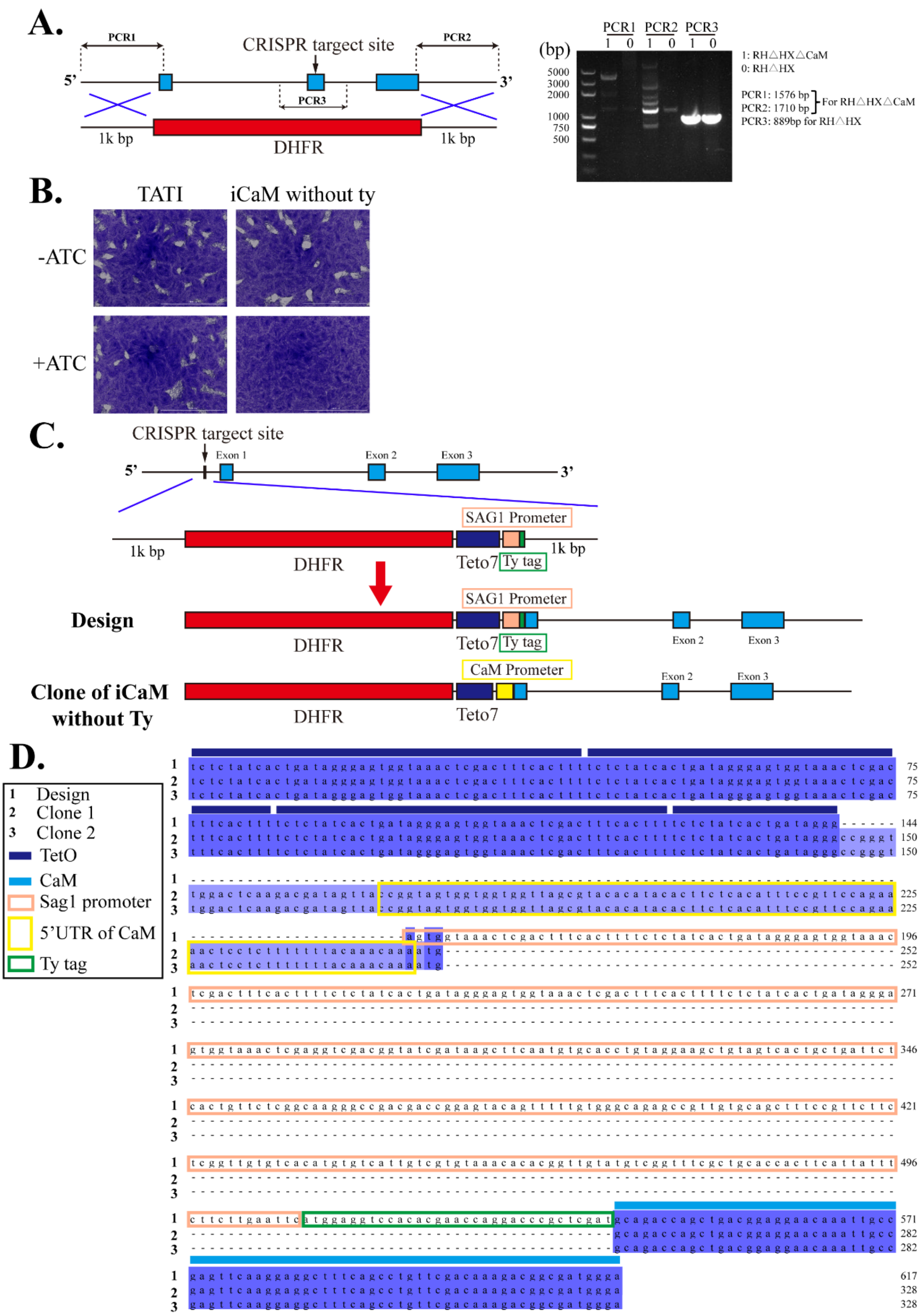
A.
PCR1
CRISPR targect site
PCR2
5'
3'
PCR3
1k bp
DHFR
1k bp
PCR1 PCR2 PCR3
1 0 1 0 1 0
(bp)
5000
3000
2000
1000
750
500
1: RH△HX△CaM
0: RH△HX
PCR1: 1576 bp
PCR2: 1710 bp
For RH△HX△CaM
PCR3: 889bp for RH△HX
B.
TATI
iCaM without ty
-ATC
+ATC
C.
CRISPR targect site
Exon 1
Exon 2
Exon 3
5'
3'
SAG1 Prometer
1k bp
DHFR
Teto7
Ty tag
1k bp
Design
SAG1 Prometer
DHFR
Teto7
Ty tag
Exon 2
Exon 3
CaM Prometer
Clone of iCaM without Ty
DHFR
Teto7
Exon 2
Exon 3
D.
1 Design
2 Clone 1
3 Clone 2
TetO
CaM
Sag1 promoter
5'UTR of CaM
Ty tag
75
75
75
144
150
150
225
225
196
252
252
271
346
421
496
571
282
282
617
328
328

Fig S2

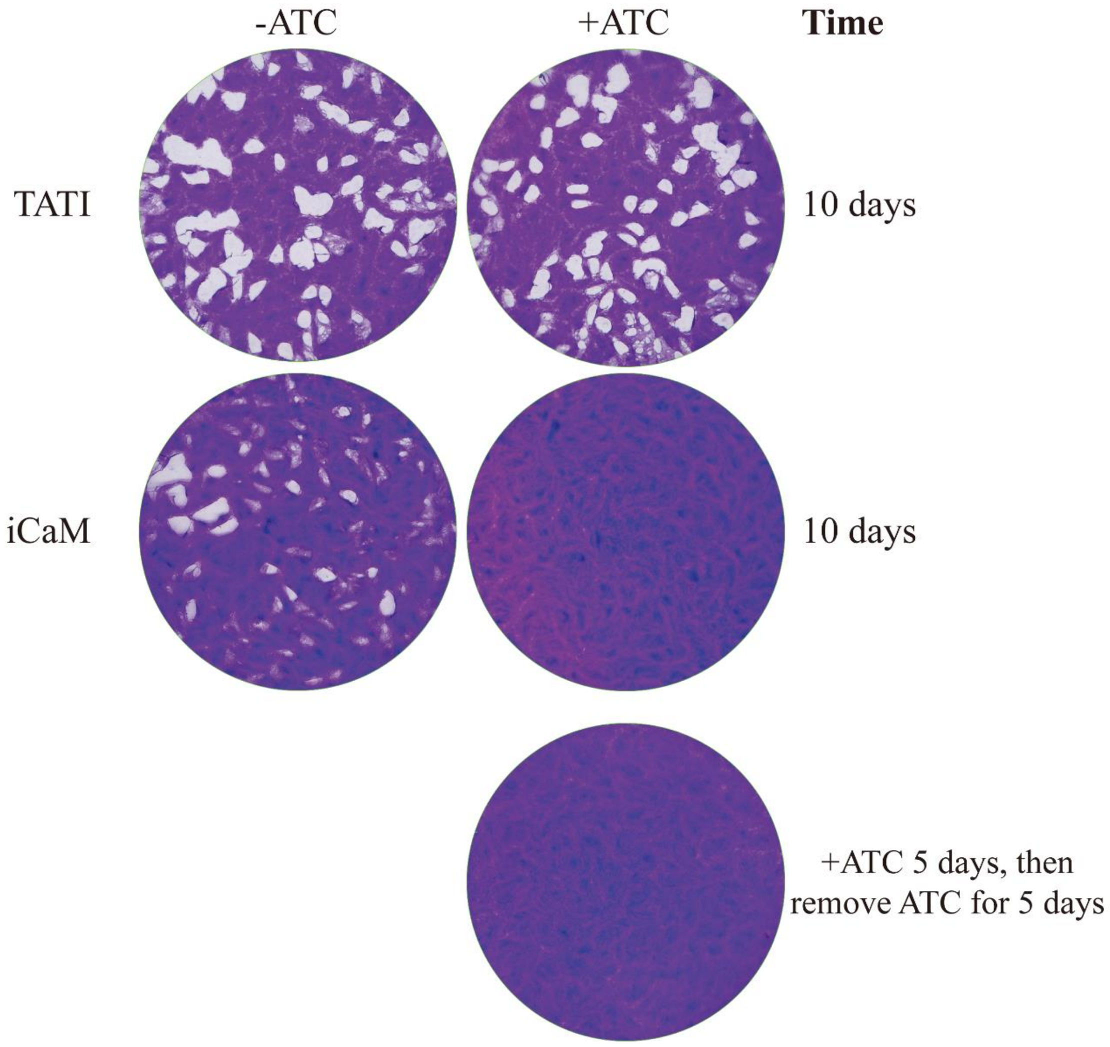

-ATC
+ATC
Time
TATI
10 days
iCaM
10 days
+ATC 5 days, then
remove ATC for 5 days

Fig S3

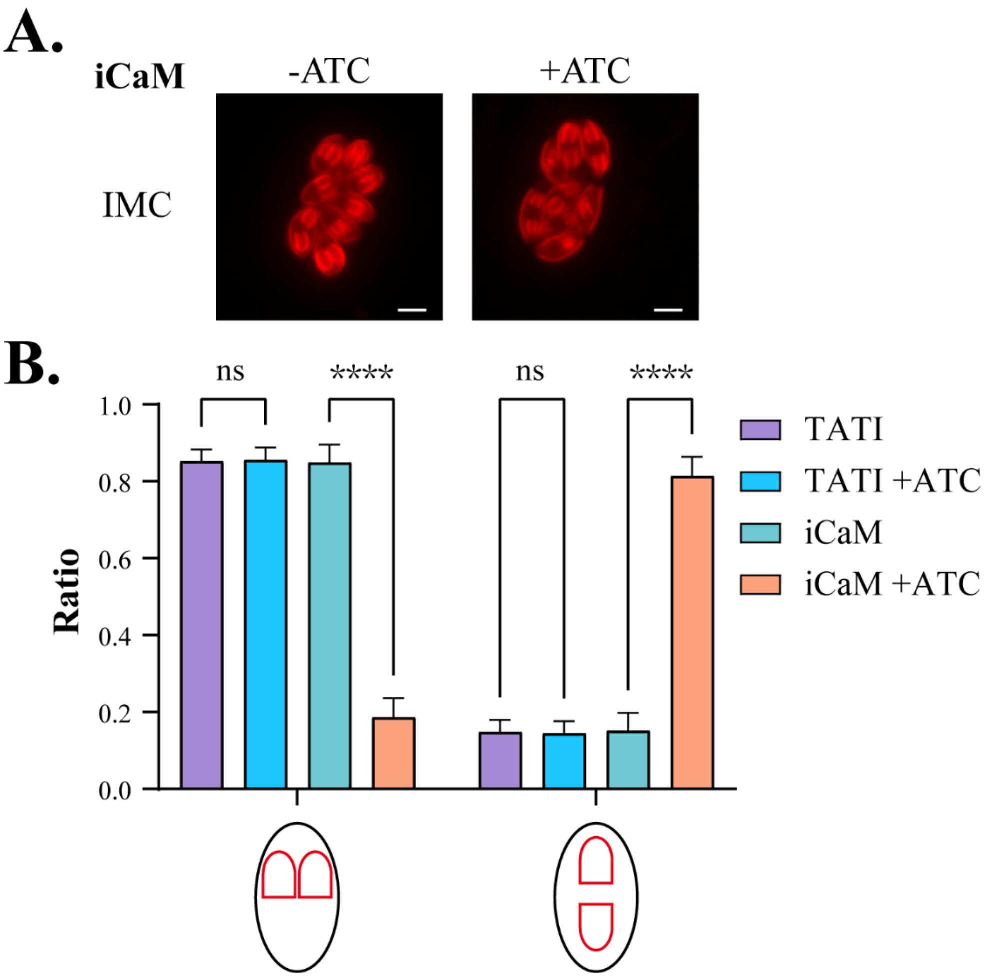
A.
iCaM
-ATC
+ATC
IMC
B.
ns
****
ns
****
1.0
0.8
0.6
0.4
0.2
0.0
Ratio
TATI
TATI +ATC
iCaM
iCaM +ATC

Fig S4

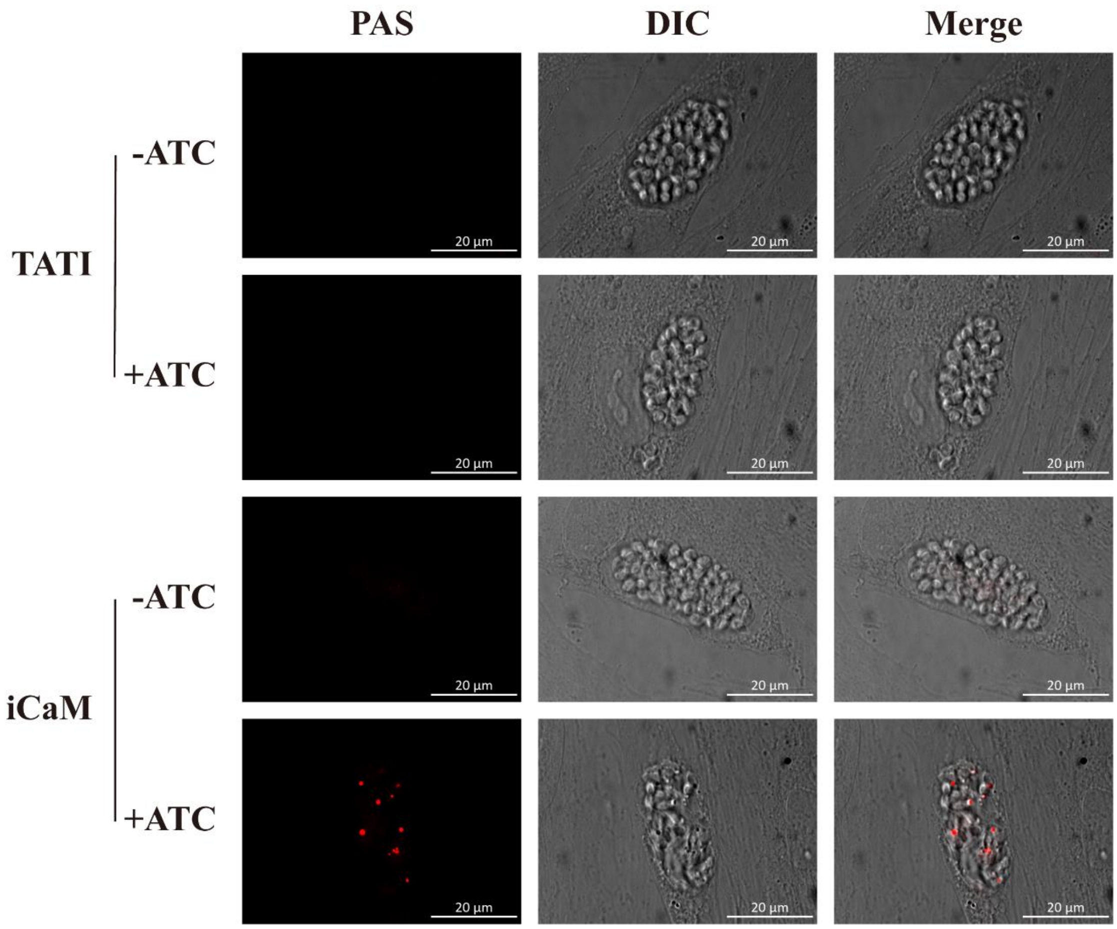

Fig S5

A. **Unique peptide count of MyoF and MyoJ identified in the CaM-BioID data**

Gene ID	Protein	CRISPR value	Molecular Weight (kDa)	Unique peptide count in CaM-BioID 1	2	3	4	Unique peptide count in Control 1	2	3	4
TGGT1_278870	Myosin F	-3.55	216	36	32	42	26	0	0	0	7
TGGT1_257470	Myosin J	-3.01	274	0	0	0	0	0	0	0	0

B.

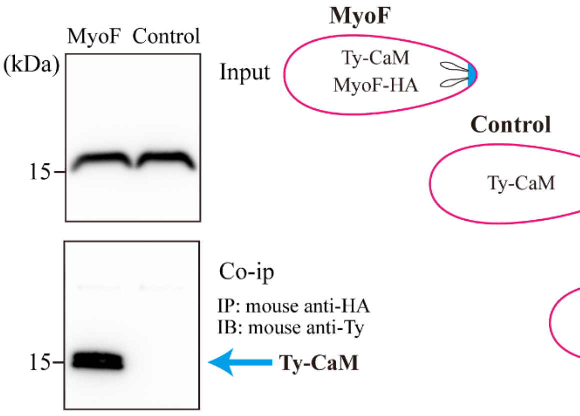

C.

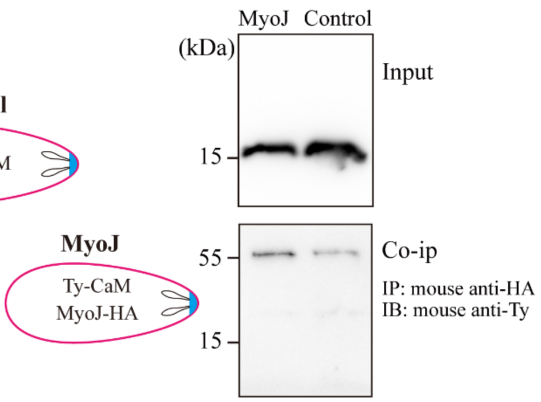

Supplement: Supplemental file 6 — Fig. S1 to S5. Download spectrum.01363-22-s0006.pdf, PDF file, 1.2 MB [file spectrum.01363-22-s0006.pdf]
